# Relationship among post-traumatic growth, spiritual well-being, and perceived social support in Chinese women with gynecological cancer

**DOI:** 10.1038/s41598-024-55605-5

**Published:** 2024-02-28

**Authors:** Yue Feng, Xingcan Liu, Shixi Zhang, Tangwei Lin, Xiujing Guo, Jing Chen

**Affiliations:** 1 Department of Gynecological Nursing, West China Second University Hospital, Sichuan University, Chengdu, Sichuan China; 2 Key Laboratory of Birth Defects and Related Diseases of Women and Children (Sichuan University), Ministry of Education, Chengdu, Sichuan China

**Keywords:** Post-traumatic growth, Spiritual well-being, Perceived social support, Gynecological cancer, Gynaecological cancer, Health care

## Abstract

This study aimed to examine the correlation between post-traumatic growth (PTG), spiritual well-being (SWB), perceived social support (PSS), and demographic and clinical factors in Chinese gynecological cancer patients. Through convenience sampling, we conducted a cross-sectional study of 771 adult patients with gynecological cancer. The European Organization for Research and Treatment for Cancer Quality of Life Questionnaire-Spiritual Well-being 32 (EORTC QLQ-SWB32), Post-traumatic Growth Inventory (PTGI), and Multidimensional Scale of Perceived Social Support (MSPSS) were used to measure SWB, PTG, and PSS. A Multiple Linear Regression Model was used to determine the possible factors contributing to PTG. The subscale with the highest centesimal score in the PTGI was the Appreciation of Life Scale, and the lowest was New Possibility. Gynecologic cancer patients with younger ages (B =  − 0.313, *P* = 0.002), perceived more family support (B = 1.289, *P* < 0.001), had more existential (B = 0.865, *P* = 0.010), and had religious belief (B = 5.760, *P* = 0.034) may have more PTG. Spiritual well-being, perceived social support, younger age, and religious beliefs are associated with post-traumatic growth in gynecological cancer patients. Healthcare staff could provide more professional support to younger patients with religious beliefs. Promoting social support and spiritual well-being could potentially serve as effective interventions for boosting PTG among gynecological cancer.

## Introduction

Gynecological cancers are severe and potentially life-threatening. There were an estimated 1.4 million new cases of gynecological cancers and more than 670 thousand deaths from gynecological cancer worldwide in 2020^1^. In China, there were more than 250 thousand newly diagnosed gynecological cancer in the same year^2^. The diagnosis of gynecological cancer and associated mortality are among the most traumatic events in women’s lives worldwide^3,4^. Diagnosed with cancer is not a discrete or single-stress experience; in contrast, cancer is an extreme chronic stressor. Unlike acute adverse events, its treatment disrupted “normal life” for a long time^5,6^. These characteristics of cancer lead people to re-examine priorities, relationships, and themselves, providing opportunities for positive psychological change^7,8^, such as post-traumatic growth (PTG). Studies have provided empirical evidence that post-traumatic growth is observed in women with gynecological cancer^9,10^.

PTG refers to a positive change that an individual experiences after various traumatic events^11^. It was found that higher PTG was associated with lower distress, a better quality of life, and prolonged survival in cancer patients^12,13^. As females with cancer have been reported to be more adversely affected by men^14^, helping gynecological cancer patients obtain more PTG is vital for improving their quality of life and mental health.

The cognitive processing theory of PTG proposed by Tedeschi^15^ regards social support as a critical environmental resource in developing PTG. Individuals with robust social support systems can better cope with significant life changes^16^. Social support has been widely interpreted as perceived support, actual receipt of social support, and satisfaction with received support^16,17^. Literature has reported that perceived social support is related to better psychological adjustment^18^. However, the mechanisms underlying the role of perceived social support in positive post-traumatic growth in patients with cancer have not been well studied, and the existing evidence on social support and post-traumatic growth remains controversial^8,19–21^. There is a need to further clarify the role of perceived social support with PTG in gynecological cancer patients.

Spirituality is another vital resource of posttraumatic growth^22^. Traumatic events can threaten an individual’s view of self and the world, necessitating the need for meaning reconstruction^23^. One way a person faces trauma and attempts to find meaning is through spirituality^23^. Previous literature has reported the important role of spiritual well-being in traumatic events^24,25^ and highlighted the positive relationship between post-traumatic growth and spiritual well-being in cancer patients^26,27^. Research has demonstrated that post-traumatic growth and spiritual well-being are concepts influenced by culture, where the cultural background significantly shapes the experience of trauma and the subsequent meaning-finding and coping mechanisms adopted by individuals^28–30^. While Chinese culture lacks formal religious development, philosophies like Buddhism, Taoism, and Confucianism have significantly shaped spiritual development and secular culture in China^28^. These philosophies instill resilience, embrace change, and encourage the pursuit of inner peace among the Chinese people^31,32^. Very few studies investigate the relationship between PTG and spiritual well-being in the Chinese context. So, this study focused on spiritual well-being as an individual resource in post-traumatic growth in Chinese.

According to Tedeschi’s^15^ theory model, we hypothesize that in the context of Chinese cultural background, perceived social support, spiritual well-being, and personal factors significantly influence post-traumatic growth in women with gynecological cancer. Therefore, this study aimed to explore the related factors affecting post-traumatic growth from perceived social support, spiritual well-being, and the patient’s demographic and clinical characters in Chinese gynecological cancer patients to help us better understand the connotation of post-traumatic growth and provide evidence for improving post-traumatic growth in cancer patients with a Chinese cultural background.

## Methods

### Participants and procedures

We conducted a cross-sectional study and continuously recruited participants from February 2020 to December 2022 in a women’s and children’s hospital. Patients were enrolled if they (1) had been diagnosed with gynecological cancer, including ovarian cancer, cervical cancer, endometrial cancer, trophoblastic tumor (malignant), and fallopian tube cancer, (2) were aged 18 years or older, and (3) were able to understand and answer relevant questions. Patients were excluded if they (1) refused to participate in the survey or (2) had cognitive impairment as cognitive challenges may affect understanding and result consistency. If patients who met the inclusion criteria were willing to participate in our study, they would provide a written consent form and cooperate with us to collect relevant information and complete questionnaires.

### Measures

#### Demographic and clinical characteristics

We collected participants’ demographic characteristics, such as age, educational background, marital status, working status, and clinical data, such as cancer type, cancer stage, diagnosis time, chemotherapy and radiotherapy history, and so on, from the medical records or self-reports of participants. We also asked whether the patients had religious beliefs or were actively engaged in spiritual practice.

#### Post-traumatic growth inventory (PTGI)

The PTGI is a 21-item scale to assess an individual’s positive changes attributed to struggling with a highly stressful event. Items were scored from 0 (no change) to 5 (great change)^33^. The total score ranges from 0 to 105, with higher scores reflecting more significant psychological growth^34^. Cronbach’s alpha for the scale in our sample was 0.954.

#### Multidimensional scale of perceived social support (MSPSS)

MSPSS is a self-report measure of subjectively assessed social support^35^. The MSPSS considers social support from three sources: family support (four items), friend support (four items), and significant others (four items). It consists of 12 items, each asking patients to answer from 1 (very strongly disagree) to 7 (very strongly agree). The total score ranges from 12 to 84, with higher scores representing higher levels of perceived social support^36^. The Cronbach’s alpha for the scale was 0.909 in the present study.

#### EORTC quality of life questionnaire-spiritual well-being32 (EORTC QLQ-SWB32)

The European Organization for Research and Treatment for Cancer Quality of Life Questionnaire-Spiritual Well-being32 was developed by the European Organization for Research and Treatment of Cancer (EORTC) based on relevant literature, expert opinions, and interviews with palliative cancer patients^37^ and validated in a multilingual and cultural context, involving a total of 14 countries including China^38^. The QLQ-SWB32 contains 32 items, with 22 of them composing four scoring scales: Existential (6 items), Relationship with Self (5 items), Relationship with Others (6 items), and Relationship with Someone or Something Greater (5 items). These items were scored from 1 (not at all) to 4 (very much). Two “skip” items for current or past belief in God or someone or something more significant, only respondents who answered yes would address the Relationship with God (one item) dimension. The rest were six non-scoring clinically relevant items^39^. The final item is used to reflect the overall SWB, which asks patients to answer from 1 (very poor) to 7 (excellent), with an additional choice of 0 (cannot reply or is unknown)^38^. The QLQ-SWB32 is a profile-based, not preference-based, measure, so these sub-scales are each scored separately; summing scales is inappropriate^38^. Total scores of the multi-item scales are transformed into centesimal scores (the score/possible highest score × 100). All scales then range from 0 to 100, with high scores representing positive outcomes. The Cronbach’s alpha values (Relationship with Others 0.737, Relationship with Self 0.762, Relationship with Someone or Something Greater 0.726, Existential 0.802) indicated good internal reliability in our sample.

### Statistical analysis

There were no missing data from our participants. Statistical analysis was performed using SPSS version 26.0 (IBM, Armonk, NY, USA). Our study presented the patients’ demographic characteristics as frequencies and percentages. The scores of the three measurement tools were tested for normality and are shown as mean, standard deviation, median, and interquartile range. Associations were first analyzed between post-traumatic growth and spiritual well-being, perceived social support, and the patient’s sociodemographic characteristics. The correlation between each subscale of post-traumatic growth and continuous variables was calculated using the Spearman correlation coefficient for the skewed data distribution. Since the scores of the PTGI did not conform to a normal distribution, the relationships between post-traumatic growth and classification variables were tested using the Nonparametric Test. Mann–Whitney U tests were used to compare two groups, and Kruskal–Wallis H tests were used for more than two groups. The Bonferroni correction was used for multiple comparisons. Multiple Linear Regression analysis was performed to identify factors affecting post-traumatic growth. Only variables with *P* < 0.05 in the correlation analysis were included. All statistical tests were two-tailed, and *P* < 0.05 was considered statistically significant.

### Ethical considerations

This study was performed in line with the principles of the Declaration of Helsinki. The hospital ethics committee approved the study protocol (code number 2019-13). The participants were informed of the study’s risks and benefits and provided written consent. The scales were completed anonymously.

## Results

### Descriptive statistics

771 patients with gynecological cancer met the eligibility criteria and participated in the study. The mean age was 48.92 ± 11.43, ranging from 18 to 74 years. Most were married (86.9%), and only 27.5% were employed. There were more than five types of gynecological cancers. 52.9% of our participants had ovarian or fallopian tube cancer, with 26.8% cervical cancer and 12.1% endometrial cancer. Most of our participants (81.3%) were diagnosed with gynecological cancer for one year and below. More than 90% of them claimed that they had no religious beliefs. Detailed information is provided in Table 1.

**Table 1:** Demographic and clinical characteristics of participants (N = 771).

Items	N	%	Items	N	%
Marriage status			Education background		
Married	670	86.9	Junior and below	405	52.5
Unmarried/divorced/widow	101	13.1	Senior middle school	161	20.9
Work status			College and above	205	26.6
Retired	208	27.0	Chemotherapy history		
Laid-off	351	45.5	Yes	497	64.5
Employed	212	27.5	No	274	35.5
Primary cancer			Radiotherapy history		
Ovarian/fallopian tube	408	52.9	Yes	69	8.9
Uterine cervical	207	26.8	No	702	91.1
Endometrial	93	12.1	Living with a partner		
Others	63	8.2	Yes	579	73.8
Stage of cancer			No	202	26.2
I	168	21.8	Family per capita monthly income (yuan)		
II	116	15.0	Less than 3000	348	45.1
III	286	37.1	3000–5000	246	31.9
IV	27	3.5	5000–10,000	129	16.7
Time after diagnosis			More than 10,000	48	6.2
1 years or below	627	81.3	Religious beliefs		
1–3 years	79	10.2	Yes	72	9.4
3–5 years	31	4.0	No	699	90.6
More than 5 years	18	2.3			

### Measurement level

Table 2 presents each measurement scale’s mean, standard deviation, median, and interquartile range. For all participants, the highest PTGI score was 51.67 on the appreciation of life scale, and the lowest was 32.12 on the new possibilities scale. The highest QLQ-SWB32 score was 78.05 on the relationship with self scale, and the lowest was 58.15 on the relationship with someone/something greater scale.

**Table 2:** Descriptive statistics for study scales and sub-scales (N = 771).

Scale	Numbers of items	Response range	Centesimal score	Mean	SD	Median	IQR
1. PTGI				43.10	24.50	41.00	37.00
Relating to others	7	0–5	41.60	14.56	8.66	14.00	13.00
New possibilities	5	0–5	32.12	8.03	5.97	7.00	9.00
Personal strength	4	0–5	45.30	9.06	5.34	9.00	8.00
Appreciation of life	3	0–5	51.67	7.75	4.29	8.00	7.00
Spiritual change	2	0–5	36.90	3.69	2.82	3.00	5.00
2. MSPSS				67.13	10.27	68.00	12.00
FaS	4	1–7	–	24.31	3.03	24.00	4.00
FrS	4	1–7	–	20.65	5.34	21.00	6.00
SO	4	1–7	–	22.17	3.92	23.00	4.00
3.QLQ-SWB32							
Existential	6	1–4	73.88	17.73	3.36	18.00	5.00
RwS	5	1–4	78.05	15.61	2.28	16.00	3.00
RwO	6	1–4	76.04	18.25	3.49	18.00	5.00
RwSG	5	1–4	58.15	11.63	2.42	12.00	3.00
Global-SWB	1	0–7	69.57	4.87	1.71	5.00	2.00

*FaS* family support, *FrS* friend support, *IQR* interquartile range, *MSPSS* multi-dimensional scale of perceived social support, *PTGI* post-traumatic growth inventory, *RwO* relationship with others, *RwS* relationship with self, *RwSG* relationship with someone/something greater, *SD* standard deviation, *SO* significant other, *SWB* spiritual well-being.

### Related factors of post-traumatic growth

We analyzed the associations between post-traumatic growth and spiritual well-being, perceived social support, and patient demographic characteristics (Tables 3, 4). The multiple linear regression analysis included variables with *P* < 0.05 (Table 5). In summary, gynecologic cancer patients who were younger (B =  − 0.313, *P* = 0.002), perceived more family support (B = 1.289, *P* < 0.001), had more existential (B = 0.865, *P* = 0.010), and had religious belief (B = 5.760, *P* = 0.034) may have more post-traumatic growth. The independent variables in this multiple linear regression analysis explained 13.0% of the model.

**Table 3:** Spearman correlation between post-traumatic growth and other continuous variable.

Variables	*r*
Family support	0.250**
Friend support	0.188**
Significant other	0.146**
Existential	0.183**
RwS	− 0.033
RwO	0.165**
RwSG	0.084*
Global-SWB	0.002
Age	− 0.194**
Time after diagnosis (month)	0.029

*RwO* relationship with others, *RwS* relationship with self, *RwSG* relationship with someone/something greater, *SWB* spiritual well-being.

*P < 0.05, **P < 0.001.

**Table 4:** Correlation between post-traumatic growth and other countable variables.

Variables	Post-traumatic growth	
	Mean (SD)	Statistics
Marriage status		1.898
Married	42.70 (24.71)	
Others	45.72 (22.94)	
Working status		6.516*
Retired	41.74 (23.72)	
Laid-off	41.49 (23.75)	
Employed	47.08 (26.08)	
Education background		24.598**
Junior and below	38.96 (23.28)	
Senior middle school	46.42 (25.29)	
College and above	48.66 (24.83)	
Primary cancer		0.886
Ovarian/fallopian tube	42.68 (24.57)	
Cervical	44.26 (24.30)	
Endometrial	41.43 (24.79)	
Stage of cancer		5.231
I	47.11 (25.02)	
II	40.40 (21.58)	
III	43.30 (25.33)	
IV	41.67 (22.95)	
Chemotherapy history		0.165
No	42.62 (24.77)	
Yes	43.36 (24.36)	
Radiotherapy history		0.034
No	43.12 (24.66)	
Yes	42.88 (22.91)	
Religious belief		5.173*
No	42.41 (24.58)	
Yes	48.78 (23.18)	
Living with a partner		0.089
No	43.22 (23.15)	
Yes	43.05 (24.98)	

^a^Mann–Whitney U tests were used for two independent samples; Kruskal–Wallis H tests were used for more than two independent samples.

^b^Multiple comparisons were corrected for significance using the Bonferroni method. The differences in the distribution of post-traumatic growth were statistically significant between *laid-off and employed* (adjusted *P* = 0.043), *junior and below, college and above* (adjusted *P* < 0.001), *junior and below,* and *senior middle school* (adjusted *P* = 0.005).

*P < 0.05, **P < 0.001.

**Table 5:** Linear regression analysis of post-traumatic growth.

Variables	B^a^	95% CI^a^	Sig.^a^	R	R^2^	Adjusted R^2^
(Constant)	3.906	− 17.841, 25.653	0.725	0.381	0.145	0.130
Age	− 0.313	− 0.508, − 0.118	**0.002**			
Family support	1.289	0.618, 1.961	** < 0.001**			
Friend support	0.188	− 0.206, 0.581	0.349			
Significant other	− 0.079	− 0.642, 0.484	0.782			
Existential	0.865	0.206, 1.525	**0.010**			
RwS	− 0.213	− 0.952, 0.526	0.571			
RwO	0.236	− 0.391, 0.863	0.460			
RwSG	0.067	− 0.747, 0.882	0.871			
Retired^b^	1.061	− 3.816, 5.939	0.669			
Employed^b^	− 1.127	− 5.584, 3.329	0.620			
Senior middle school^b^	4.081	− 0.482, 8.644	0.080			
College and above^b^	4.450	− 0.236, 9.137	0.063			
Religious belief	5.760	0.450, 11.070	**0.034**			

*RwS* relationship with self, *RwO* relationship with others, *RwSG* relationship with someone/something greater.

^a^Unstandardized coefficients beta with 95% confidence interval for B.

^b^The working status and education background was converted to three dummy variables.

## Discussion

This study explored the factors contributing to post-traumatic growth in Chinese patients with gynecological cancer. Our findings confirmed our hypothesis of posttraumatic growth in cancer survivors that factors promoting PTG could be categorized according to the environmental resource (perceived social support), individual resource (spiritual well-being), and personal factors (clinical characteristics: age and religious belief). The present sample reported a median of 43.10 on the total score of post-traumatic growth, which was lower than the results of Zhou^9^ (56.50) in Chinese gynecological cancer patients. One possible reason is that their study subjects were patients who received chemotherapy after surgery, whereas our study included perioperative gynecological cancer patients. The study by Wang^40^ reported that patients with a more extended time since diagnosis were likelier to have higher PTG. Therefore, there may be more newly diagnosed cases in our study. We found that the subscale with the highest score in the PTGI was the appreciation of life, which is consistent with Zhou. When facing the traumatic event of gynecological cancer, patients can reevaluate their own life values and gain a different perspective on the meaning of life. The lowest score in the PTGI in our study was the new possibilities subscale. It is also a relatively low dimension in Zhou’s results^9^. One possible reason is that the average age of our participants is around 50 years old, which may not be as energetic as younger women who are willing to explore entirely new lifestyles. Additionally, most participants had been diagnosed within one year, and their primary focus post-diagnosis was on how to treat the illness. There might be a lack of contemplation regarding establishing life pursuits, setting new goals, and trying new experiences.

We found that perceived social support was positively correlated with post-traumatic growth among Chinese gynecological cancer patients. The result is consistent with many prior studies. For example, a longitudinal study of 206 long-term cancer survivors found a significant association between perceived social support and posttraumatic growth^41^. A study among 344 Chinese women with gynecological cancer indicated that women with higher perceived social support tended to experience more post-traumatic growth^9^. Perceived social support refers to many types of assistance, including emotional, instrumental, and financial, provided by a network of individuals such as family, friends, and significant others in times of need^42^. In contrast to received social support, perceived social support refers to the available social support one believes they can acquire from their social network^43^. According to the literature, social support can assist women with gynecological cancer to better comprehend their situation by providing more opportunities to express their trauma and survival^44^. In our study, family support was found to impact post-traumatic growth. This is likely because a family-oriented approach, with the Chinese emphasizing interdependence and obligations to family members^45^ characterizes Chinese culture. As a result, family members are an essential source of social support^34^ and support from family members may help the Chinese more than non-family members^45^. Therefore, to enhance the post-traumatic growth of gynecological cancer patients in China, future intervention measures could aim at improving various sources of support, especially family support. For instance, promoting shared positive beliefs within the family regarding the illness and treatment, strengthening interaction among family members, assisting in sharing caregiving responsibilities for cancer patients, and fostering internal support within the family^46^.

This study also indicated a positive correlation between spiritual well-being and post-traumatic growth, consistent with previous studies^26,27^. Spiritual well-being is one of the most critical aspects of human existence, as it provides a driving force that gives a person stability, meaning, fulfillment in life, and faith in self^47^. In the Chinese cultural context, religious beliefs such as Buddhism, Taoism, or Christianity, traditional Chinese philosophies such as Confucianism and Daoism, which emphasize a tranquil, tolerant, and harmonious attitude towards life^48,49^, as well as spiritual seeking, can all serve as sources of support for individuals facing trauma. Family and social support emphasized in traditional Chinese culture, certain spiritual practices like meditation and prayer, as well as individuals’ perceptions and acceptance of destiny^31,32^, may contribute positively to post-traumatic growth. This correlation reflects the profound impact of cultural, belief, and societal factors on individual spiritual well-being, emphasizing the importance of considering spiritual factors within the context of cancer care and mental health practices.

As for demographic characteristics, we found that patients who were younger and had religious beliefs reported significantly higher levels of PTG. Our findings were consistent with a study from Sim^50^, which reported that age was negatively associated with post-traumatic growth. Younger people may have a longer life expectancy, or they have more and stronger planning abilities for their current and future lives, such as work, family, and children. Moreover, compared with younger patients, older people may often be under pressure from other major life events^51^. Therefore, more supportive care can be provided for elderly cancer patients to promote their post-traumatic growth. This may include professional mental health services, facilitating family interaction and social activities among elderly patients, and providing comforting spaces for spiritual well-being. The result of religion being an influencing factor of PTG in our study was in line with previous literature that religious patients may experience more PTG^50,52^. The use of religion as a coping approach in cancer patients could be regarded as a means for finding meaning in life-threatening trauma events^50^.

### Limitations

Although this study has significant clinical implications, its limitations must be noted when interpreting the results. Because the study had a cross-sectional design with a convenience sample, no causal association between the variables was found. Future studies can be longitudinally designed to capture the changes and developments between social support, spiritual well-being, and post-traumatic growth. It helps to reveal the dynamic relationship between these variables rather than just a static correlation. Most of our subjects had a disease duration of one year or less, and we failed to reach more terminal patients who may have had more thinking about spirituality. Therefore, our findings may not necessarily apply to such patients. Furthermore, compared to people from other cultures, some concepts, such as support from friends and significant others, may have different meanings for Chinese people. Whether these findings can be applied to other cultures should be determined. Finally, the results of this study might have limited generalizability due to convenience sampling. Future studies should consider multi-centered investigations for improved robustness.

### Clinical implications

This research was conducted in the context of no formal religious development. In certain studies, spiritual well-being has been linked to religious faith; however, this should not be confused with religion^53,54^. Spirituality is a broad term that can be experienced by anybody, regardless of religious faith. As a result, clinical staff should pay close attention to cancer patients’ spiritual well-being, and spiritual care should be considered an integral component of cancer treatment. For example, healthcare professionals can incorporate questions about spiritual well-being in nursing assessment to gain a more comprehensive understanding of patients’ needs. In clinical practice, we could inform patients about spiritual support and resources, such as places of solace, and ensure they know the available spiritual support within the healthcare team. Additionally, there is a need to train healthcare professionals to deliver spiritual care to patients. This may involve considering patients’ spiritual needs in treatment plans, offering supportive conversations, or coordinating care activities related to patients’ spirituality.

Our results suggest that promoting post-traumatic growth by improving the perceived social support and spiritual well-being of gynecological cancer patients may be possible. However, the correlation between the various dimensions of perceived social support and spiritual well-being with post-traumatic growth remains complex. Further studies should explore the interaction between these variables, such as employing mediation analysis or structural equation modeling. Alternatively, qualitative studies could be conducted to explore how patients with cancer achieve post-traumatic growth through social support and spiritual well-being. This may help clinical staff better understand the relationship and promote this growth. In addition, healthcare professionals can incorporate critical social resources from the family into existing spiritual therapy interventions to design culturally appropriate psychosocial interventions better.

Furthermore, healthcare professionals should receive appropriate training to proficiently recognize and foster post-traumatic growth in cancer patients. This training encompasses specialized knowledge, covering concepts related to PTG, assessment tools, and coping strategies. Proficiency in communication skills is equally vital, allowing healthcare professionals to employ supportive and empathetic language during patient interactions and encouraging them to share their experiences. Promoting interdisciplinary teamwork, involving doctors, nurses, social workers, and other professionals in collaborative supportive care for patients, is crucial.

## Conclusions

In conclusion, our study revealed that women with gynecological cancer who display more PTG might likely be younger, have religious beliefs, and experience more perceived social support and spiritual well-being. Our findings propose some new opportunities for promoting post-traumatic growth in gynecological cancer patients in the Chinese context. To enhance the post-traumatic growth of gynecological cancer patients in China, more supportive care can be provided for cancer patients who are elderly and have religious beliefs. Future intervention measures could improve various sources of support, especially family support, and provide patients with spiritual care.

## Data Availability

The datasets used and analyzed during the current study are available from the corresponding author upon reasonable request.

## References

[1] Sung H, Ferlay J, Siegel RL, Laversanne M, Soerjomataram I, Jemal A, Bray F (2021). Global cancer statistics 2020: GLOBOCAN estimates of incidence and mortality worldwide for 36 cancers in 185 countries. CA Cancer J. Clin..

[2] Cao W, Chen H-D, Yu Y-W, Li N, Chen W-Q (2021). Changing profiles of cancer burden worldwide and in China: A secondary analysis of the global cancer statistics 2020. Chin. Med. J..

[3] Gonen G, Kaymak SU, Cankurtaran ES, Karslioglu EH, Ozalp E, Soygur H (2012). The factors contributing to death anxiety in cancer patients. J. Psychosoc. Oncol..

[4] Tonsing KN, Vungkhanching M (2018). Assessing psychological distress in cancer patients: The use of distress thermometer in an outpatient cancer/hematology treatment center. Soc. Work Health Care.

[5] Mehnert A, Koch U (2010). Prevalence of acute and post-traumatic stress disorder and comorbid mental disorders in breast cancer patients during primary cancer care: A prospective study. Psycho-oncology.

[6] Sumalla E, Ochoa C, Blanco I (2009). Posttraumatic growth in cancer: Reality or illusion?. Clin. Psychol. Rev..

[7] Marziliano A, Tuman M, Moyer A (2020). The relationship between post-traumatic stress and post-traumatic growth in cancer patients and survivors: A systematic review and meta-analysis. Psycho-Oncology.

[8] Scrignaro M, Barni S, Magrin ME (2011). The combined contribution of social support and coping strategies in predicting post-traumatic growth: A longitudinal study on cancer patients. Psycho-Oncology.

[9] Zhou LH, Hong JF, Qin RM, Henricson M, Stenmarker M, Browall M, Enskar K (2021). Post-traumatic growth and its influencing factors among Chinese women diagnosed with gynecological cancer: A cross-sectional study. Eur. J. Oncol. Nurs..

[10] Jewett PI, Vogel RI, Galchutt P, Everson-Rose SA, Teoh D, Radomski M, Blaes AH (2022). Associations between a sense of connection and existential and psychosocial outcomes in gynecologic and breast cancer survivors. Support. Care Cancer.

[11] Tedeschi RG, Calhoun LG (1996). The posttraumatic growth inventory: Measuring the positive legacy of trauma. J. Traum. Stress.

[12] Dougall AL, Swanson J, Kyutoku Y, Belani CP, Baum A (2017). Posttraumatic symptoms, quality of life, and survival among lung cancer patients. J. Appl. Biobehav. Res..

[13] Walsh D, Morrison TG, Conway RJ, Eamonn R, Sullivan FJ, Annmarie G (2018). A model to predict psychological- and health-related adjustment in men with prostate cancer: The role of post traumatic growth, physical post traumatic growth, resilience and mindfulness. Front. Psychol..

[14] Messelt A, Thomaier L, Jewett PI, Lee H, Teoh D, Everson-Rose SA, Blaes AH, Vogel RI (2021). Comparisons of emotional health by diagnosis among women with early stage gynecological cancers. Gynecol. Oncol..

[15] Tedeschi RG, Calhoun LG (2004). Posttraumatic growth: Conceptual foundations and empirical evidence. Psychol. Inq..

[16] Thoits PA (1982). Conceptual, methodological, and theoretical problems in studying social support as a buffer against life stress. J. Health Soc. Behav..

[17] Thoits PA (1995). Stress, coping, and social support processes: Where are we? What next?. J. Health Soc. Behav..

[18] Hefner J, Eisenberg D (2009). Social support and mental health among college students. Am. J. Orthopsychiatry.

[19] Prati G, Pietrantoni L (2009). Optimism, social support, and coping strategies as factors contributing to posttraumatic growth: A meta-analysis. J. Loss Trauma.

[20] Widows MR, Jacobsen PB, Booth-Jones M, Fields KK (2005). Predictors of posttraumatic growth following bone marrow transplantation for cancer. Health Psychol..

[21] Yeung NCY, Lu Q (2018). Perceived stress as a mediator between social support and posttraumatic growth among Chinese american breast cancer survivors. Cancer Nurs..

[22] Czyzowska N, Raszka M, Kalus A, Czyzowska D (2021). Posttraumatic growth and spirituality in mothers of children with pediatric cancer. Int. J. Environ. Res. Public Health.

[23] Cadell S, Regehr C, Hemsworth D (2003). Factors contributing to posttraumatic growth: A proposed structural equation model. Am. J. Orthopsychiatry.

[24] Nooripour R, Hosseinian S, Hussain AJ, Annabestani M, Maadal A, Radwin LE, Hassani-Abharian P, Pirkashani NG, Khoshkonesh A (2021). How resiliency and hope can predict stress of covid-19 by mediating role of spiritual well-being based on machine learning. J. Relig. Health.

[25] Nooripour R, Hosseinian S, Sobhaninia M, Ghanbari N, Hassanvandi S, Ilanloo H, Kakabraee K (2022). Predicting fear of COVID-19 based on spiritual well-being and self-efficacy in Iranian university students by emphasizing the mediating role of mindfulness. Pract. Clin. Psychol..

[26] Gesselman AN, Bigatti SM, Garcia JR, Coe K, Cella D, Champion VL (2017). Spirituality, emotional distress, and post-traumatic growth in breast cancer survivors and their partners: An actor-partner interdependence modeling approach. Psychooncology.

[27] Sinclair S, Booker R, Fung T, Raffin-Bouchal S, Enns B, Beamer K, Ager N (2016). Factors associated with post-traumatic growth, quality of life, and spiritual well-being in outpatients undergoing bone marrow transplantation: A pilot study. Oncol. Nurs. Forum.

[28] Feng Y, Liu X, Lin T, Luo B, Mou Q, Ren J, Chen J (2021). Exploring the relationship between spiritual well-being and death anxiety in patients with gynecological cancer: A cross-section study. BMC Palliat. Care.

[29] Fekih-Romdhane F, Riahi N, Achouri L, Jahrami H, Cheour M (2022). Social support is linked to post-traumatic growth among Tunisian postoperative breast cancer women. Healthcare.

[30] Nooripour R, Ghanbari N, Hosseinian S, Ronzani TM, Hussain AJ, Ilanloo H, Majd MA, Soleimani E, Saffarieh M, Yaghoob V (2022). Validation of the spiritual well-being scale (SWBS) and its role in predicting hope among Iranian elderly. Ageing Int..

[31] Yang C-T, Narayanasamy A, Chang S-L (2012). Transcultural spirituality: The spiritual journey of hospitalized patients with schizophrenia in Taiwan. J. Adv. Nurs..

[32] Shiah YJ, Chang F, Tam WCC, Chuang SF, Yeh LC (2013). I don’t believe but I pray: Spirituality, instrumentality, or paranormal belief?. J. Appl. Soc. Psychol..

[33] Lianchao A, Tingting M (2020). Mindfulness, rumination and post-traumatic growth in a Chinese cancer sample. Psychol. Health Med..

[34] Zhang L, Lu Y, Qin Y, Xue J, Chen Y (2020). Post-traumatic growth and related factors among 1221 Chinese cancer survivors. Psycho-Oncology.

[35] Zimet GD, Dahlem NW, Zimet SG, Farley GK (1988). The multidimensional scale of perceived social support. J. Person. Assess..

[36] Liu J, Xia LX (2018). The direct and indirect relationship between interpersonal self-support traits and perceived social support: A longitudinal study. Curr. Psychol..

[37] Vivat B, Young T, Efficace F, Sigurdadottir V, Arraras JI, Asgeirsdottir GH, Bredart A, Costantini A, Kobayashi K, Singer S (2013). Cross-cultural development of the EORTC QLQ-SWB36: A stand-alone measure of spiritual wellbeing for palliative care patients with cancer. Palliat. Med..

[38] Vivat B, Young TE, Winstanley J, Arraras JI, Black K, Boyle F, Bredart A, Costantini A, Guo J, Irarrazaval ME (2017). The international phase 4 validation study of the EORTC QLQ-SWB32: A stand-alone measure of spiritual well-being for people receiving palliative care for cancer. Eur. J. Cancer Care.

[39] Rohde Gudrun E, Young T, Winstanley J, Arraras Juan I, Black K, Boyle F, Bredart A, Costantini A, Guo J, Irarrazaval Maria E (2019). Associations between sex, age and spiritual well-being scores on the EORTC QLQ-SWB32 for patients receiving palliative care for cancer: A further analysis of data from an international validation study. Eur. J. Cancer Care.

[40] Wang J, She Y, Wang M, Zhang Y, Lin Y, Zhu X (2021). Relationships among hope, meaning in life, and post-traumatic growth in patients with chronic obstructive pulmonary disease: A cross-sectional study. J. Adv. Nurs..

[41] Schroevers MJ, Helgeson VS, Sanderman R, Ranchor AV (2010). Type of social support matters for prediction of posttraumatic growth among cancer survivors. Psycho-Oncology.

[42] Razieh N, Seyed Saeed N, Marzieh M (2019). Post-traumatic growth among family caregivers of cancer patients and its association with social support and hope. Int. J. Community Based Nurs. Midwif..

[43] Feng D, Su S, Wang L, Liu F (2018). The protective role of self-esteem, perceived social support and job satisfaction against psychological distress among Chinese nurses. J. Nurs. Manag..

[44] Pinar G, Okdem S, Buyukgonenc L, Ayhan A (2012). The relationship between social support and the level of anxiety, depression, and quality of life of Turkish women with gynecologic cancer. Cancer Nurs..

[45] You J, Lu Q (2014). Sources of social support and adjustment among Chinese cancer survivors: Gender and age differences. Support. Care Cancer.

[46] Wang Q, Sheng Y, Wu F, Zhang Y, Xu X (2019). Effect of different sources support on adaptation in families of patient with moderate-to-severe dementia in China. Am. J. Alzheimer’s Dis. Other Dementias.

[47] Alorani OI, Alradaydeh MTF (2018). Spiritual well-being, perceived social support, and life satisfaction among university students. Int. J. Adoles. Youth.

[48] Dong M, Wu S, Zhu Y, Jin S, Zhang Y (2017). Secular examination of spirituality–prosociality association: Survey research in nonreligious-based populations in China. Arch. Psychol. Relig..

[49] Zhu J, Wong FKY, Zhu J, Wong FKY (2021). Global case study in spirituality: Stories of hope from China. Spiritual Dimensions of Advanced Practice Nursing.

[50] Sim BY, Lee YW, Kim H, Kim SH (2015). Post-traumatic growth in stomach cancer survivors: Prevalence, correlates and relationship with health-related quality of life. Eur. J. Oncol. Nurs..

[51] Cormio C, Romito F, Giotta F, Mattioli V (2015). Post-traumatic growth in the Italian experience of long-term disease-free cancer survivors. Stress Health.

[52] Kim Y, Kim Y, Kwak Y (2021). Factors associated with post-traumatic growth in male patients with rectal cancer: A cross-sectional study. Eur. J. Oncol. Nurs..

[53] Gonzalez P, Castaneda SF, Dale J, Medeiros EA, Buelna C, Nunez A, Espinoza R, Talavera GA (2014). Spiritual well-being and depressive symptoms among cancer survivors. Support. Care Cancer.

[54] Johnson KS, Tulsky JA, Hays JC, Arnold RM, Olsen MK, Lindquist JH, Steinhauser KE (2011). Which domains of spirituality are associated with anxiety and depression in patients with advanced illness?. J. Gen. Intern. Med..

